# An Overview of the Current Scientific Evidence on the Biological Properties of *Abelmoschus esculentus* (L.) Moench (Okra)

**DOI:** 10.3390/foods14020177

**Published:** 2025-01-08

**Authors:** Carsten Tsun-Ka Kwok, Yam-Fung Ng, Hei-Tung Lydia Chan, Shun-Wan Chan

**Affiliations:** 1 Department of Food and Health Science, Technological and Higher Education Institute of Hong Kong, Hong Kong, China; tsun-ka-carsten.kwok@connect.polyu.hk (C.T.-K.K.); yfng@cuhk.edu.hk (Y.-F.N.); lydiachan@thei.edu.hk (H.-T.L.C.); 2 Department of Food Science and Nutrition, The Hong Kong Polytechnic University, Hong Kong, China; 3 University Safety Office, The Chinese University of Hong Kong, Hong Kong, China

**Keywords:** *Abelmoschus esculentus* (L.) Moench, okra, pharmacology, antihyperlipidemic, antidiabetic, antifatigue

## Abstract

*Abelmoschus esculentus* (L.) Moench, commonly known as okra or lady’s finger, is an annual flowering plant belonging to the *Malvaceae* family. Okra is a native plant in Africa as well as a traditional medicine in Africa and India for treating different diseases and conditions. Today, okra is widely consumed as a vegetable and is increasingly recognized as a superfood due to its rich nutritional profile and potential pharmacological benefits. Research indicates that okra exhibits a range of biological activities, including antidiabetic, antihyperlipidemic, antifatigue, vasoprotective, hepatoprotective, antitumor, anti-inflammatory, and antimicrobial effects. Despite its promising therapeutic potential, research on the active compounds in okra and evaluating efficacy in clinical settings remains limited. This review aims to consolidate existing scientific knowledge on the biological and pharmacological properties of okra, thereby encouraging further investigation into its health benefits. Ultimately, this could pave the way for the development of functional foods or health supplements that leverage okra as a key ingredient to prevent chronic diseases and enhance overall health outcomes.

## 1. Introduction

The global prevalence of chronic diseases is on the rise. A multinational survey study has demonstrated a significant increase in the percentage of teenagers aged 11 to 17 years with four or more chronic disease risk factors, soaring by approximately 30% from 2003–2007 to 2013–2017 [1]. Consequently, there has been a heightened demand for functional foods as the public becomes increasingly conscious of their consumption. Beyond providing essential nutrition, these foods can play a vital role in mitigating the development of chronic diseases and enhancing overall well-being [2,3]. Vegetables like moringa and turmeric are widely recognized as functional foods with a diverse range of pharmacological effects, including enhancing fertility and alleviating various chronic conditions such as cardiovascular diseases, diabetes, obesity, inflammatory bowel disease (IBD), acne, asthma, eczema, and allergies, supported by both clinical and preclinical studies [4,5]. Unlike moringa and turmeric, okra is an emerging functional food that has been known for its antidiabetic, antihyperlipidemic, and antifatigue effects. However, currently, there is no evidence to support the use of okra in inflammatory diseases such as IBD, asthma, and mastitis, even though okra possesses anti-inflammatory effects and antioxidative effects [6]. Therefore, further research is essential to uncover the full extent of okra’s biological activity.

**Figure 1:**
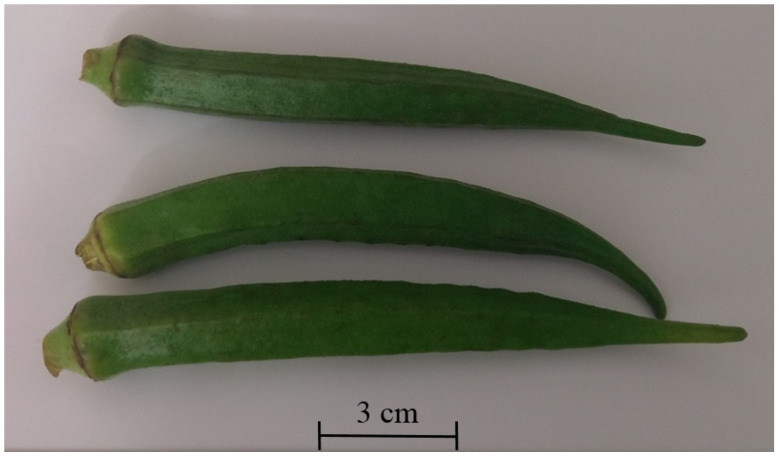
Fruit of okra.

**Figure 2:**
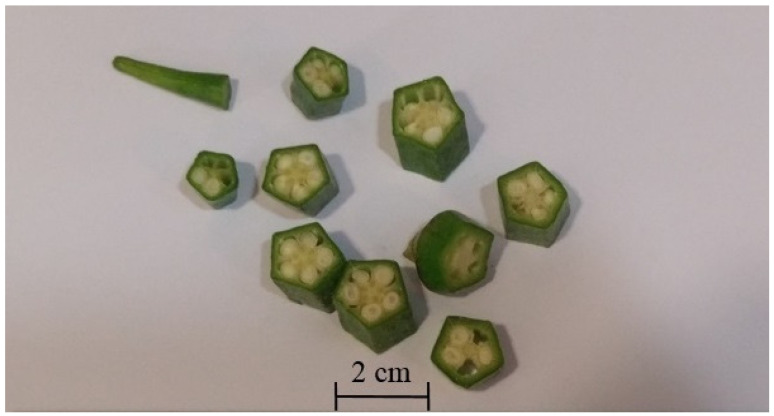
Cross section of okra fruit with seeds.

The scientific name of okra is *Abelmoschus esculentus* (L.) Moench (Figure 1 and Figure 2). It is also known as lady’s finger, as well as gumbo. This perennial flowering plant belongs to the family of Malvaceae. Its origin is still under debate. The majority believes that it is from Africa, probably Ethiopia (Sudan), instead of India [7]. Today, okra is cultivated worldwide in the tropics, subtropics, and warm regions like South Asia (China, India, etc.), Europe, and Australia, as well as the Americas (the United States and Brazil), and is extensively consumed as a vegetable globally, especially in Africa [7,8]. Meanwhile, okra is recorded as a traditional medicine in India and Africa, for instance, in Ghana [9,10]. Traditionally, the okra pod is used to treat sexually transmitted diseases (gonorrhea and syphilis), urinary diseases (ardor urine and dysuria), dysentery, muscle spasms, catarrh, fever, diarrhea, constipation, anemia, dermal disease (pruritus), and even as a cosmetic product (lotion). It has also been used as a cordial, sudorific (to promote sweating), and aphrodisiac, with historical records suggesting its efficacy in preventing scurvy [8,11,12,13,14,15,16,17,18,19].

Okra, recognized as a superfood (functional food), is increasingly gaining recognition for its high nutritional value and diverse therapeutic effects, which are supported by scientific evidence [20,21]. Furthermore, okra’s easy availability in the market is a notable advantage. Due to its abundant cultivation, okra remains affordably priced, making it a desirable functional food option [6]. Although the consumption of okra is becoming popular, currently, there is no review summarizing both clinical and preclinical data of okra supporting its usage in different diseases. This review aims to provide an overview of the currently available scientific information on okra in both preclinical and clinical studies to draw attention from researchers to studying undiscovered biological activities of okra, its active components, and the investigating the efficacy of okra in different diseases in clinical trials. Ultimately, this could pave the way for the development of functional foods or health supplements that leverage okra as a key ingredient to prevent chronic diseases and enhance overall health outcomes.

## 2. Active Ingredients and Nutrition Value in Okra

Okra stands out as a functional food due to its exceptional nutritional profile. It is rich in essential nutrients, boasting a significant carbohydrate content (7 g per 100 g serving), protein (2 g per 100 g serving), dietary fiber (3.2 g per 100 g serving), an array of minerals (abundant in potassium, calcium, phosphorus, and manganese), and vitamins, while being low in fat (0.1 g per 100 g serving) [22,23] (Table 1).

A total of 35 active components have been isolated from various parts of okra, primarily from the pods and seeds. Among these components, the majority are flavonoids (16 in total) and polysaccharides (12 in total) [24,25,26,27,28,29,30]. These active components, along with their biological effects and sources of isolation, are summarized in Table 2.

## 3. Biological Activities of Okra

Okra has been reported to possess a wide range of biological activities, including antidiabetic, antihyperlipidemic, antifatigue, antitumor, and immunomodulating properties [46,57,58,59]. This section will provide a comprehensive overview of these biological activities and their underlying mechanisms (Table 3 and Table 4).

### 3.1. Antidiabetic Effect

Restoration of β-cell function, improvement in insulin resistance or sensitivity through suppression of peroxisome proliferator-activated receptor (PPAR)-γ, and enhancement of antioxidant enzymes, as well as scavenging of free radicals, inhibition of glucose absorption, retardation of carbohydrate digestion, reducing blood glucose levels, and improving glucose tolerance are the crucial working principles underlying the antihyperglycemic effect of *Abelmoschus esculentus* (L.) Moench fruit, seeds, and peel [38]. The detailed mechanisms of okra’s antidiabetic effect will be discussed as follows.

#### 3.1.1. Restoration of β-Cell Function

The protective effect of *Abelmoschus esculentus* (L.) Moench on pancreatic islets, particularly β-cells, has become one of the key targets of recent research. Okra fruit extract has been found to reverse the streptozotocin-induced β-cells damage and prevent free fatty acid-induced apoptosis of β-cells [61,86]. For example, an in vivo study found that administration of okra fruit extract (200 mg/kg) significantly suppressed insulin levels, the homeostasis model assessment of basal insulin resistance (HOMA-IR), as well as blood glucose levels in streptozotocin-induced diabetic rats [61]. These changes might be associated with the increase in the mass of pancreas islets and the number of β-cells in diabetic rats, which was proposed to play a key role in the restoration of β-cells function [61]. Similarly, subfractions of okra fruit also showed improved glycemic control in a high-fat diet and streptozotocin-induced diabetes in rats [60]. Although subfraction 1 (F1: rich in quercetin glucosides, such as isoquercetin and pentacyclic triterpene ester) and subfraction 2 (F2: rich in polysaccharides and carbohydrates) could significantly lower blood glucose levels, HOMA-IR, and glycated hemoglobin (HbA1c), and the effects of F2 are more effective than F1. The preventative effect of okra on β islet damage was related to the antihyperglycemic effect [60], which can be further supported in vitro in the RINm5f cell line with palmitate-induced β-cell apoptosis, which demonstrates that F1 and F2 prevented free fatty acid-induced β-cell apoptosis significantly through the downregulating expression of dipeptidyl peptidase-4 (DPP-4) apoptotic signaling and restoring the expression level of glucagon-like peptide-1 receptor (GLP-1R) [86]. Both F1 and F2 decreased in the sub-G1 stage through the downregulation of the expression of pro-caspase 3 and active-caspase 3, suppressing DPP-4, as well as modulating palmitate-induced signal cascades (the one that causes β-cell apoptosis) via the downregulation of adenosine monophosphate-activated protein kinase (AMPK) and Bax, as well as the upregulation of the mammalian target of rapamycin (mTOR) and phosphoinositide 3-kinase (PI3K). However, the effect of F2 on the downregulation of AMPK and suppression of cascades is more significant than F1 [86].

#### 3.1.2. Improvement in Insulin Resistance/Sensitivity via Suppression of PPARs Genes

Apart from the restoration of β-cell function, okra has also been shown to improve insulin sensitivity through the downregulation of PPARs gene expression. Several studies discovered that okra, particularly its polysaccharides, were antagonists of PPARs, which ameliorated insulin resistance and insulin sensitivity.

An in vivo study showed that the amelioration in insulin resistance/sensitivity in high-fat diet-induced diabetes in rats relied on the effect of okra fruit extract suppressing mRNA levels of PPAR-α and -γ in the pancreas [61]. These findings were aligned with the one in the mice with high-fat diet-induced obesity, which demonstrated that ethanol extract from okra alleviated insulin resistance via the downregulation of mRNA levels of PPAR-α and -γ in the liver (caused by obesity) significantly [25]. Similarly, okra fruit polysaccharide significantly attenuated the expression of PPAR-α, -γ, and -β/δ in adipose tissue in the mice [49].

#### 3.1.3. Enhancement of Antioxidant Enzymes as Well as Scavenging of Free Radicals

Increasing evidence has shown that oxidative stress plays a crucial role in the development of diabetes. Excessive production of free radicals [reactive oxygen species (ROS)/reactive nitrogen species (RNS)] and weakened antioxidant defenses can cause oxidation of macromolecules and cell damage, particularly β-cells [98,99]. Studies found that okra seeds, peel, and fruit possess strong antioxidant activity and enhance antioxidant defense systems in diabetic rats [58,64]. Therefore, the ability of okra to free radical scavenging effects and restoration of the antioxidant enzyme system also plays an essential role in its antidiabetic effects.

A study investigated the in vivo antioxidant activity in okra seeds and peel, which found that okra significantly increased antioxidant enzyme levels, including superoxide dismutase (SOD), catalase (CAT), glutathione peroxidase (GPx), and reduced glutathione (GSH), as well as attenuated lipid peroxidation in the liver, pancreas, and kidney [58]. Additionally, another study showed that okra fruit also possessed excellent in vivo antioxidant activity (the ferric-reducing ability of plasma assay); it decreased the activity of erythrocyte plasma membrane redox system (PMRS), erythrocyte malondialdehyde (MDA) content (prevent lipid peroxidation), and advanced oxidation protein products (AOPP) (hinder protein oxidation); as well as increased erythrocyte GSH [64].

The okra flower, fruit, leaf, and seed (methanol extracts/enrichment fraction of water extracts) also demonstrated good scavenging free radical in both 2,2-diphenyl-1-picrylhydrazyl (DPPH) and ferric-reducing antioxidant power assays. The results also indicated that there was a positive proportional relationship between phenolic content, flavonoid content, and antioxidative activities [87]. Similarly, another study further indicated that phenolic compounds, including procyanidin B2, procyanidin B1, catechin, epicatechin, quercetin, and rutin (okra seeds do not contain catechin and epicatechin, while pulp does not have quercetin and procyanidin B2) might be the active molecules responsible for antioxidant activity in okra [37]. Moreover, four flavonoid compounds in okra fruit, 5,7,3′,4′-tetrahydroxy-4″-O-methyl flavonol-3-O-β-D-glucopyranoside, 5,7,3′,4′-tetrahydroxy flavonol-3-O-[β-D-glucopyranosyl-(1→6)]-β-D-glucopyranoside, isoquercitrin, and quercetin 3-O-gentiobioside, showed high antioxidant activity [25,27]. Last but not least, a pectic polysaccharide WOP-2, which is a rhamnogalacturonan I with type-II arabinogalactan side-chains (580KDA composed with monosaccharides Rha (21.4%), GalA (34.9%), Gal (29.6%), GlcA (4.5%), Glc (5.9%), and Ara (3.7%) was identified. It had strong free radical scavenging activity in a dose-dependent manner and was shown to boost antioxidant enzyme (SOD) levels in diabetic mice, which prevented damage in β-cells caused by peroxidation and helped restore insulin levels [40].

Okra was also found to possess a therapeutic effect on gestational diabetes in rats through suppressing oxidative stress and insulin resistance, which is achieved by restoration of antioxidant defense, such as SOD, GPx, GSH, and CAT, in the liver and pancreas [65].

The antioxidant activity in okra and its active ingredients, epically isoquercitrin and quercetin-3-O-gentiobiose, not only contributed to its antidiabetic effect but was also found to be attributed to its hepatoprotective effect, antifatigue effect, vasoprotective effect, and neuroprotective effect (for instance, reducing the risk of developing Alzheimer’s disease) [31,33,73,92].

#### 3.1.4. Inhibition of Rate of Carbohydrate Digestion and Glucose Absorption

The antidiabetic effects of okra were also found, depending on the retardation of the rate of starch digestion and glucose absorption. In vitro studies have shown that aqueous extract from the okra peel and seeds inhibited α-glucosidase and α-amylase activities appreciably in a dose-dependent manner [38,88]. The effect of okra peel was more potent than its seeds [88]. In unripe seeds, oligomeric proanthocyanidins, which are composed of epigallocatechin and catechin extension units, were inhibitors of α-glucosidase and α-amylase [38]. However, another study found that rutin and quercetin 3-gentiobioside are also active compounds responsible for suppressing carbohydrate digestion [32].

In an in vivo study, the water-soluble fraction (dietary fiber) of okra fruit was able to reduce the intestinal absorption of glucose significantly in fasting rats. Interestingly, when okra and metformin were fed to diabetic rats, the effect of metformin on intestinal absorption of glucose vanished [54]. The effect of okra reduction in intestinal absorption of glucose was found to be concentration-dependent in an in vitro study [89]. These results suggested that okra is useful for postprandial glucose control.

#### 3.1.5. Hypoglycemia and Improving Glucose Tolerance

The antidiabetic effects of okra also relied on the fact that it lowered fasting blood glucose levels and improved glucose tolerance. Okra fruit, seeds, and peel were found to lower blood glucose levels and HbA1c considerably in different models of diabetic rats, which were either induced by alloxan or streptozotocin [66,67].

Okra polysaccharides from its fruit were demonstrated to reduce blood glucose levels and improve glucose tolerance in mice with high-fat diet-induced obesity [49]. Isoquercitrin and quercetin 3-O-gentibiosidein in okra were responsible for the hypoglycemic effect of okra in high-fat diet-induced obesity in mice [25]. Meanwhile, a polysaccharide, rhamnogalacturonan, was identified and responsible for lowering blood glucose levels and improving glucose tolerance in diabetic mice [28].

#### 3.1.6. Prevention of Diabetic Nephropathy

An in vitro study demonstrated that fractional extract from okra fruit, especially F1 and F2, could improve diabetic nephropathy through inhibition of diabetic renal epithelial to mesenchymal transition (EMT), and the regulation of DPP-4 and GLP-1R, as well as reducing oxidative stress and renal fibrosis in the HK-2 cell line [90]. The same study showed that F1 was rich in pentacyclic triterpene and flavonoid glycosides, such as quercetin glycosides. In contrast, F2 was mainly composed of polysaccharides of uronic acid, galactose, glucose, and myo-inositol [90].

The effect of F1 and F2 on relieving diabetic nephropathy was found to be achieved by modifying the signal involved in developing EMT. F1 significantly suppressed high glucose-induced increased levels of vimentin, angiotensin II receptor-1 (AT-1), and transforming growth factor β1 (TGF-β1), as well as DPP-4 activity and upregulated high attenuated levels of cadherin. Similarly, F2 has almost the same effect as F1 except for no significant change in the level of TGF-β1 [90]. Similarly, in vivo studies also found that both F1 and F2 could ameliorate diabetic nephropathy, where the effect of F2 was much more specific to the kidney. Even though both fractions could improve renal function and alleviate renal fibrosis, only F2 was able to reverse the DPP-4 and GLP-1R levels as well as attenuate oxidative stress in the kidney [69].

### 3.2. Antifatigue and Vasoprotective Effect

Recent studies suggested that okra possesses antifatigue properties, which might enhance exercise tolerance by reducing the accumulation of metabolic by-products, increasing energy reserves, and regulating energy metabolism. Additionally, okra has been shown to mitigate oxidative stress by modulating enzymatic activities involved in energy metabolism and the excitation–contraction coupling process.

An in vivo study showed that okra ethanol extract and its polysaccharides could alleviate fatigue in mice. Okra polysaccharides and ethanol extract enhanced exercise endurance in a dose-dependent manner via lowering blood lactic acid (BLA), as well as serum urea nitrogen (SUN), and increasing the hepatic glycogen (HG) notably, in which the effect of the polysaccharides was much better than the extract. The polysaccharides could also improve kidney function in mice with kidney yang deficiency [57]. Another study also found that two okra polysaccharide fractions, AEP-1 and AEP-2, possessed antifatigue activity in accordance with the previous study [71]. The same study also found that okra polysaccharides could increase muscle glycogen (MG), and the effect of AEP-1 was stronger than AEP-2. Regarding the mechanistic pathways of AEP-1 and AEP-2, their effects were related to the enhancement of the removal of BLA by decreasing the content of lactate dehydrogenase (LDH), decreasing creatine kinase (CK) in blood and improving energy metabolism via increasing succinate dehydrogenase (SDH), adenosine 5′-triphosphatase (ATPase), and energy content (ATP) in the serum, liver, and muscle in three different states (resting, dynamic, and recovery states) [71].

Other research also found that the okra seed in the pod was the part responsible for the antifatigue effect of okra, and the result aligned with the aforementioned studies. This study revealed that okra seeds significantly improved antioxidant defense enzymes (SOD and GSH-Px) and scavenge free radicals. The flavonoid compounds in okra seeds, particularly isoquercitrin and quercetin 3-O-gentiobiose, were likely to be responsible for their antifatigue activity because of their antioxidant activity [31]. Another investigation found that quercetin 3-O-gentiobiose relieved fatigue significantly by increasing gastrocnemius muscle glycogen [33].

In addition, quercetin 3-O-gentiobiose also possesses a vasoprotective effect by preventing exhaustive exercise-induced vascular endothelial dysfunction by improving aortic morphology, preventing oxidative stress damage, and suppressing inflammation. Quercetin 3-O-gentiobiose reduced the number of foam cells and aorta thickness, as well as intima–media thickness in the exhaustive swimming rats. This was due to its high antioxidant enzyme activities, its effect on decreasing inflammatory cytokines monocyte chemoattractant protein-1 (MCP-1), tumor necrosis factor alpha (TNF-α), and interleukin-6 (IL-6) significantly, and dose-dependently, its modulating effect on the LOX-1/NF-κB signaling pathway, which remarkably reduced mRNA expression and protein expression of lectin-like oxidized low-density lipoprotein receptor-1 (LOX-1), intercellular adhesion molecule-1 (ICAM-1), and nuclear transcription factor-κB p65 (NF-κB p65) expressions in a dose-dependent manner [33].

### 3.3. Hepatoprotective Activity

A few studies found that okra pods and roots had a hepatoprotective effect via their excellent antioxidant activity and their ability to boost the enzymatic antioxidant defense system. In vivo and in vitro studies showed that okra roots reversed the hepatic damage induced by carbon tetrachloride (CCl_4_) and restored its function in HepG2 cells and rats’ livers, as okra significantly prevented the leakage of alanine transaminase (ALT), aspartate transaminase (AST), and alkaline phosphatase (ALP), lowered the level of total bilirubin, increased serum albumin, prevented accumulation of triglyceride in the liver, and improved histopathology of the liver, as well as reduced levels of TNF-α in the liver (preventing immune-mediated liver injury) [72].

Another in vivo study in rats also demonstrated the pre-treatment of rats with ethanol extract from okra pods exhibited a hepatoprotective effect, which prevented the elevation of some liver health-related biomarkers, such as serum glutamate oxaloacetate transaminase (GOT), serum glutamate pyruvate transaminase (GPT), ALP, and gamma-glutamyltransferase (GGT), as well as an increase in cholesterol and triglycerides. But unlike okra root, okra ethanol extract could not lower the level of bilirubin. It could also suppress liver inflammation and increase hepatic total protein as well as non-protein sulfhydryls [73].

Quercetin 3-O-gentiobiose and quercetin 3-O-glucosyl (1→6) glucoside isolated from okra seeds were the active compounds in okra pods for the hepatoprotective effect. These flavonoids were shown to ameliorate hepatic damage mediated by CCl_4_ [26]. These compounds can serve as antioxidants to scavenge ROS and upregulate endogenous antioxidant enzyme levels (CAT, GSH, and SOD) to prevent CCl_4_-induced oxidative stress and CCl_4_-induced lipid oxidative stress, as evidenced by a decrease in the level of MDA and an increase in CAT, GSH, and SOD levels [72,73].

### 3.4. Antihyperlipidemic Activity

Investigations found that different parts of okra (peel, seeds, and pods) could alter dyslipidemia in mice and rats, and some improvements are even comparable to the effect of the lipid-lowering medication simvastatin [60,61,67,74]. Dyslipidemia is a well-known risk factor for developing obesity that could lead to diabetes and cardiovascular disease [100,101]. Therefore, okra may be used as a dietary source for preventing these diseases. In vivo studies showed that okra seed and peel powder reversed a high-fat diet or a high-fat diet plus streptozotocin-induced abnormal lipid profile (total triglycerides, total cholesterol, and low-density lipoprotein) in rats [61,67]. Similarly, subfractions of okra extract F1 (rich in flavonoid and quercetin glycosides) and different okra extracts also suppressed high-fat diet plus streptozotocin-induced as well as tyloxapol-induced hyperlipidemia in rats and mice [60]. However, ethanol extract from okra alleviated high-fat diet-induced hepatic steatosis and macrovesicular steatosis in C57BL/6 mice, in which isoquercitrin and quercetin 3-O-gentiobioside were found to be the active compounds [25]. Apart from improving lipid profit, okra polysaccharides could also reduce the size of white adipocytes in high-fat diet-induced obesity in C57BL/6 mice [49].

The underlying mechanism of okra and its active components in antihyperlipidemic activity was revealed by these studies, showing that okra extract reduced the transcription of lipogenesis and cholesterol metabolism-related genes as well as nuclear receptor transcription factors, such as PPARs, Liver X receptors (LXR), LXR, and PPARs target genes, and adipocyte protein 2 (aP2) [25,61]. Additionally, okra polysaccharides inhibited the gene expression of LXR α/β in liver and adipose tissue, ATP-binding cassette transporter G1 (ABCG1), Apolipoprotein E (ApoE), cytochrome P450 7A1 (CYP7A1), and lipoprotein lipase (LPL), as well as PPARs (γ, α, and β/δ) in adipose tissue and mitochondrial uncoupling protein 2 (UCP2) [49]. Okra also promoted the fecal excretion of bile acid via the upregulation of the transcription of CYP7A1, while the downregulation of the transcription of the sterol regulatory element-binding protein 1c (SREBP1c) and fatty acid synthase (FAS) were also accounted for okra’s hypolipidemic activity [75].

### 3.5. Antitumor Activity

Various components in okra and its compounds have the ability to impede the advancement of cancer cells by inducing apoptosis, inhibiting proliferation, and causing cell cycle arrest. Additionally, the immunomodulatory properties of different components in okra may also play a role in its antitumor activity.

The lectin isolated from okra seeds showed antiproliferation and apoptosis in human breast cancer cells (MCF7) but not in skin fibroblast (CCD-1059 sk). The selective antitumor activity (cytotoxic) of lectin on MCF7 relied on its interaction with carbohydrates on the cell surface [29]. The underlying mechanism of lectin-induced apoptosis in MCF7 was mediated by the upregulation of apoptosis-related gene expression, including caspase-3 and -9, as well as p21 and the downregulation of Bcl-2 transcription, which increased the ratio of Bax to Bcl-2 r. However, no alteration was found in the survivin, apoptosis-inducing factor (AIF) and endonuclease G gene [29].

Pectic rhamnogalacturonan-I (RG-I) extracted from okra pods retarded proliferation and induced apoptosis in B16F10 Melanoma cells in the tPs culture plate and the one cultured in anti-adhesive polyHEMA substratum (3D). This was mediated by arresting the cell cycle (increased cells in the G2/M phase dramatically) as well as decreasing the protein expressions of cadherins and α5 integrin, as well as upregulating galectin-3 (Gal-3) [41].

Polysaccharides isolated from different parts of okra possessed immunomodulatory activity by promoting the maturation of dendritic cells (DCs cells), modulating cytokine secretion, and activating macrophages [76,91]. For instance, polysaccharide extract from okra fruit stimulated primary cell-rat bone marrow hematopoietic cells derived immature dendritic cells (BMHC-imDCs), which was proved by the upregulation of major histocompatibility complex (MHC) class II and Cluster of differentiation (CD) 80/86 and decreasing endocytosis activity dose-dependently. The activation of DCs increased the secretion of IL-12/ interferon gamma (IFN-γ) and decreased the secretion of IL-10. This indicated okra could trigger a type 1 T helper (TH1) response [91]. Another study showed that a water-soluble polysaccharide (OFPS11) from okra flowers could suppress the proliferation of HepG-2 cells with the aid of the immunomodulatory effect of OFPS11 on the RAW264.7 cell, which is primarily composed of galactose and rhamnose in 2.23:1 ratio [46]. The immunomodulatory effect of OFPS11 significantly increased the phagocytic activity in the macrophages in a dose-dependent manner, as well as its production of nitric oxide (NO), TNF-α, and IL-1β. These increases were caused by the upregulation of mRNA and protein expressions of inducible nitric oxide synthase (iNOS), TNF-α, and the activation of the NF-κB signaling pathway [46]. The research evaluated the immunomodulatory effect of okra polysaccharides [RPS, composed of galactose (40%), rhamnose (29.9%), galacturonic acid (13.9%), and glucuronic acid (9.4%)] and its purified fractions RPS-1 [principally consisted of galactose (33.1%), galacturonic (31.9%), and rhamnose (20%)], RPS-2 [mainly consisted of galactose (35.5%), galacturonic (31.4%), and rhamnose (20.3%)] and RPS-3 [primarily composed of galacturonic (25.1%), galactose (21.6%), galacturonic (17.8%), glucose (14.9), and rhamnose (1.8)] in vitro in RAW264.7 and RPS2 in vivo in BALB/c mice. The RPSs showed the same result as the OFPS11 in increasing NO secretion through the upregulation of iNOS in the in vitro study. The PRSs also increased the secretion of cytokines, such as TNF-α (for all RPSs), IFN-γ (for RPS-1), and IL-10 (for all RPSs), while RPS-2 significantly increased splenocyte proliferation and thymus and spleen index in vivo [76].

### 3.6. Neuroprotective Effect

Oxidative stress and psychological stress could cause the development of neurodegenerative diseases, such as Alzheimer’s disease (AD) [102,103]. Aqueous and methanol extract from okra seeds was found to have anti-stress and nootropic (attenuation of scopolamine-induced cognitive impairment) effects in an in vivo study (elevated plus maze task and forced swimming test (FST) was employed for anti-stress, while passive avoidance was used to determine nootropic effect) as well as demonstrated antioxidant effects [77]. Furthermore, another in vivo study also showed okra seeds and leaves have fair antidepressant activity (FST and tail suspension test) dose-dependently [78]. As a result, okra may mitigate neurodegenerative diseases and their symptoms.

An in vivo study revealed that pre-treatment of ethanol extract from okra and its flavonoid compounds (quercetin and rutin) had a neuroprotective effect and improved cognitive impairment in dexamethasone-treated ICR male mice [30]. The same study showed the pre-treatment significantly improved the performance of mice in the Morris water maze test, mitigated the morphological damage in the cornu ammonis 3 (CA3) region of the hippocampus, and reversed the decreased number of CA3 hippocampal neurons, as well as increased the average number of Brdu-positive cells per section in the histology. It also increased the expression of NR (NMDA-receptor) 2A/B protein remarkably. This indicated that pre-treatment of okra could reverse the damage in the hippocampus through enhancement of cell proliferation in the dentate gyrus (in the CA3 region) and recover the number of N-methyl-D-aspartate (NMDA) receptors [30]. Okra was once again proven to be beneficial to neurodegenerative disease. Similarly, an in vitro study revealed that ethanolic extract from okra could reduce the risk of development of AD or other neurodegenerative diseases, especially in people who express the H63D variant in the hemochromatosis (HFE) gene in the neuroblastoma SH-SY5Y cell line [92]. The same study reported that okra significantly attenuated oxidative stress (lower protein carbonyl, H_2_O_2_, and intracellular ROS), suppressed tau phosphorylation at serine 199, 202, and 396 in a dose-dependent manner, and inhibited the activity of glycogen synthase kinase-3 beta (GSKk-3β) by increasing serine 9. The mechanism behind this was believed to be related to the decrease in the intercellular iron level.

### 3.7. Skin Protective Effect

Okra has a historical tradition of use in cosmetics. Presently, okra seed extract has been utilized as the active ingredient of a commercial cosmetic product. An in vivo study indicated that okra significantly improved skin elasticity, firmness, texture, and density, as well as mitigated wrinkles, which was related to the protective effect of okra seeds on fibroblast growth factor-2 (FGF-2) stimulating cell proliferation and glycosaminoglycans (GAG) synthesis [80]. Another study demonstrated that okra had the potential as sunscreen, as flavonoids enrichment of okra could alleviate ultraviolet radiation-B induced oxidative stress and cytotoxicity in human dermal fibroblast adult cells (HDFs) by its good antioxidant effect in an in vitro study and intracellular ROS assay as well as its promoting effect on enzymatic antioxidant defense [SOD, CAT, GPx, and glutathione reductase (GR)] probably via reducing protein expressions of nuclear factor E2-related factor-2 (Nrf2) and hemeoxygenase-1 (HO-1) significantly in a dose-dependent manner [93].

### 3.8. Relief Temporomandibular Joint (TMJ) Inflammatory Hypernociception Through Its Anti-Inflammatory, Antinociceptive, and Analgesic Activity

An in vivo study found that methanolic and water extracts of okra peel possess great anti-inflammatory, analgesic, and antinociceptive activities [81]. Another study also showed that lectin (20.0 kDa) extracted from okra seeds exhibited good antinociceptive and anti-inflammatory activities [52]. Due to the discovery of antinociceptive, anti-inflammatory, and analgesic activities of lectin, recently, the efficacy of lectin from okra seeds and its involved pathways were examined in TMJ inflammatory hypernociception in rats.

In the zymosan-induced TMJ inflammatory hypernociception in rats, pre-treatment with okra lectin could lower leukocyte cell, myeloperoxidase (MPO) activity, and Evans blue dye extravasation in the synovial lavage, as well as decrease inflammatory cell influx in synovial membrane significantly. It could also lower the mechanical hypernociception in rats (less head withdrawal) as well as decrease the cytokines levels in TMJ tissue and trigeminal ganglion, including IL-1β and TNF-α, which contribute to inflammation and nociception [82]. On the other hand, okra lectin also demonstrated similar results in the formalin-induced TMJ inflammatory hypernociception model [83].

The possible molecular mechanisms of okra lectin were elucidated by these studies. Its effects were found to be mediated by the HO-1 pathway (increase HO-1 expression) but not iNOS, as well as the activation of central opioid receptors (δ and κ but not µ) [82,83].

### 3.9. Anti-Gastric Ulcer Effect of Okra via Its Gastroprotective Effect and Anti-Adhesive Effect of Helicobacter pylori on the Gastric Epithelial Cells

Recently, an in vivo study reported that pre-treatment with okra demonstrated a strong gastroprotective effect on the ethanol-induced model, which could improve the histology of gastric mucosa significantly (edema, hemorrhage, and inflammation scores), decrease oxidative stress (lower MDA and retention of GSH), and increase cell proliferation in the healing area [84].

Several studies found that pre-treatment with okra fruit extract, for instance, as aqueous extract with human gastric epithelia AGS cells, possessed an anti-adhesive effect on *Helicobacter pylori* (*H. pylori*), in which some of the active compounds/molecules were identified. An in situ study stated that crude polysaccharides with a rhamnogalactan backbone have strong anti-adhesive activity towards *H. pylori*. This effect is due to its acid subfraction of polysaccharide (AF-III with a galacturonans backbone consisting of uronic acid clusters and glucuronic acid content) and glycoprotein fraction [48]. Another study further identified that the responsible polymer in the crude polysaccharide for the anti-adhesive effect on *H. pylori* was acetylated rhamnogalacturonan-I polymers [50]. The mechanism of the anti-adhesive effect of okra on *H. pylori* was agreed to be the non-specific interaction between compounds/molecules of okra, like polysaccharides, and binding factors/sites of *H. pylori,* such as SabA, Laminin, lactoferrin, BabA, HpA, and fibronectin (interaction with which binding factor is unknown) [50,94]. Moreover, it is suspected that the charge of the molecules might influence the non-specific interaction [94]. Furthermore, the acetylation/esterification of rhamnogalacturonan-I polymers was necessary for its anti-adhesive effect on *H. pylori* [50]. Interestingly, a study found that the anti-adhesive effect of okra on *H. pylori* with outer membrane protein Q genotype 1 (HopQ type 1) was better than the one with either both HopQ type 1 and 2 or HopQ type 2; it also worked well on *H. pylori* with cytotoxin-associated gene A (CagA) [95]. Apart from the anti-adhesive effect on *H. pylori*, it has also been demonstrated that the methanolic extract from okra possesses bacteriostatic and bactericidal effects against clinical isolates of *H. pylori.*

It is well known that gastric ulcers can be caused by alcoholic consumption and infection with *H. pylori*. The ability of okra to prevent alcohol-induced gastric injury and the gastric attachment of *H. pylori* makes okra a new potential strategy for the amelioration of gastric ulcers. This is because the effectiveness of first-line treatment of *H. pylori*-induced gastric ulcers utilizing antibiotics is usually low due to poor bioavailability to the inner layers of gastric mucosa and the emergence of antibiotic resistance [104]. However, further investigation is required to validate the efficacy of okra in gastric ulcers.

### 3.10. Antimicrobial Activity

Various research studies found that okra exhibits antibacterial properties and an antifungal effect. Specifically, palmitic and stearic acids were the active compounds responsible for its antimicrobial effects [24,96].

An in vitro study showed that lyophilized and freshwater extracts from the okra pods significantly inhibited bacterial growth, including *Rhodococcus opacus*, *Mycobacterium* sp., *M. aurum*, *Staphylococcus aureus*, and *Xanthobacter Py2*, as evidenced by minimum inhibitory concentration (MIC) and disk diffusion [24]. The same study revealed that okra extracts suppressed the cell viability of these bacterial strains and that the antibacterial effect was not related to the alteration of bacterial protein (catalase) and denaturation of DNA. Furthermore, it revealed that the polar lipids fraction of okra (rich in palmitic acid and stearic acid) was responsible for its antibacterial effect. Another in vitro study showed that methanolic extract from okra pods significantly inhibited the growth of different clinical isolates of *H. pylori* and had a potent bactericidal effect on *H. pylori* BAA009, *H. pylori* BAA026, and *H. pylori* ATCC 43504, but the exact mechanism was not revealed [97]. Similarly, an in vitro study demonstrated that okra seeds significantly inhibited the growth of *Listeria monocytogenes*, *Salmonella enteritidis*, and *S. typhimurium* [96]. The same study also reported that okra possessed significant fungistatic and fungicidal effects on *Aspergillus fumigatus* and *A. ochraceus*, and the effects were superior to the positive control, ketoconazole.

## 4. Clinical Evidence of Okra

In recent years, there have been around 10 clinical studies investigating the efficacy and safety of okra, mainly focusing on glycemic control and lipid profile in patients with type 2 diabetes and diabetic nephropathy; however, some of them showed contradicted results [105,106,107,108,109,110,111,112,113,114] (clinical studies’ findings were summarized in Table 5). For instance, a clinical study showed that 1000 mg powdered okra supplement three times per day for three months could significantly improve glycemic control and hyperlipidemia in diabetic patients in Iran (lowering TG and TC) [113]. In contrast, another study revealed that a 1000 mg powdered okra capsule could remarkably improve glycemic control but not lipid profile in diabetic patients in Iran receiving oral hypoglycemic medication [111]. Similarly, one clinical study supported the administration of two 500 mg okra powder capsules three times per day for eight weeks, which significantly alleviated hyperlipidemia and reduced liver and kidney damage (lowering ALT, AST, and uric acid) in prediabetic patients [105]. Additionally, other studies showed that an 80 mg dried okra extract capsule per day for 10 days did not have a significant effect on renal function and lipid profile in patients with diabetic nephropathy [106,110]. The conflicting results may stem from variations in dosage and duration of the intervention. Despite the inconsistency in findings from clinical studies, meta-analyses have supported the safety of consuming okra, which can notably enhance glycemic control. Additionally, consuming ≤3000 mg/day (powdered okra) has been shown to alleviate hyperlipidemia [115].

A novel formula known as IQP-AE-103, comprising a dehydrated powder of okra pods and inulin, [116] showed a significant effect on reducing body weight and body fat in overweight and moderately obese subjects [114]. This clinical study offers promising evidence for the potential use of okra in managing obesity, warranting further clinical investigations to validate its efficacy.

## 5. Perspectives

Even though okra is widely consumed as food or folk medicine, the pharmacological research on it is still preliminary. Because most of the studies still examine the effect of crude extract or fraction extract from okra on its pharmacological effect, particularly on its antidiabetic effect, preventing EMT, antifatigue effect, antihyperlipidemic activity, immunomodulatory activities, anti-gastric ulcer effect, and antimicrobial effect, as well as skin protection effect. This might result from the sticky mucilage in okra hindering the isolation of bioactive molecules, or there was insufficient investigation of active components from the okra stem, flower, and leaf [42,117]. Future studies should aim to optimize extraction methods to isolate active compounds, especially polysaccharides. Additionally, more research is needed to investigate compounds isolated from the okra stem, flower, and leaf that may be responsible for the pharmacological effects of okra. For instance, identifying the specific compound responsible for modulating PPARs and improving β-cell apoptosis would be a valuable area for further exploration.

The pharmacological effects of okra have not been well studied, particularly regarding the antifatigue effect, anti-gastric ulcer effect, and antimicrobial effect. More mechanistic studies are needed to understand these effects. For example, currently, the study of the antimicrobial effect of okra mainly focused on its antibiotic activity, it will be worth studying its effects on host response, such as how it controls bacterial infection. In vitro studies showed that enhancing macrophage phagocytosis and intracellular killing of bacteria by nitric oxide and ROS in *S. aureus*-infected macrophages effectively remove *S. aureus* infections [118,119]. Hence, future studies could explore the effect of okra in *S. aureus*-infected macrophages. Additionally, some of the traditionally claimed pharmacological effects of okra, such as anti-scorbutic, anemia, aphrodisiac, cordial, and sudorific, lack scientific support and require further investigation. Although okra demonstrated hyperlipidemic activity, its beneficial effects on cardiovascular disease and non-alcoholic fatty liver disease (NAFLD) remain unknown and warrant examination in future studies. Furthermore, inflammatory diseases like mastitis and IBD share similar pathogenesis involving inflammation, oxidative stress, and compromised epithelial barrier [120,121]. Okra may ameliorate these conditions due to its anti-inflammatory effect and protective effect on epithelial cells and ability to suppress oxidative stress. Thus, investigating the effects of okra on inflammatory diseases in future studies may be worthwhile.

Current clinical evidence on the pharmacological effects of okra is limited, with most clinical studies focusing on okra’s efficacy in improving glycemic control and lipid profile in patients with type 2 diabetes, diabetic nephropathy, or prediabetes. Since okra demonstrated significant effects on alleviating diabetes and hyperlipidemia in clinical trials, future clinical trials may consider investigating the efficacy of okra on CVD, obesity, and NAFLD as these diseases share similar pathogenesis, such as impaired blood glucose and hyperlipidemia and are interconnected [122]. Although okra showed significant improvement in lipid profile and glycemic control in clinical studies (Table 5), it is worth mentioning that these clinical trials are mainly conducted in Iran and suggested daily consumption of ≤3000 mg of okra powder. These results may lack diversity in sociodemographics, particularly race and ethnicity, which might lead to poor generalizability and applicability of trial outcomes in diverse patient groups [123]. Therefore, future clinical studies studying the efficacy of okra in different diseases should involve diverse sociodemographic groups and optimize the daily dose of okra consumption to maximize its beneficial effect.

Apart from the direct consumption of okra to obtain its beneficial effect, there are new supplements and food products that incorporate okra as a functional ingredient, allowing the public to maintain physical well-being. For instance, a formula, IQP-AE-103, composed of dehydrated powder from okra pods and inulin, has been proven effective in controlling weight in obese subjects [114]. Similarly, okra seed flour has been incorporated into rice noodles with tapioca starch, which showed improved glycemic control in healthy individuals [124]. Furthermore, research studies demonstrated that okra polysaccharide and okra pectin have good emulsification performance and stability [125,126]. In addition, okra mucilage was reported to be a good replacement for fat in ice cream [127]. Therefore, there is likely to be an increase in food (potentially cake and salad dressings) incorporating okra as a functional ingredient.

Potential interactions between okra and other standard medications for chronic diseases, particularly diabetes, should be investigated, as a study showed that okra diminished the absorption of metformin in rats [54]. Conversely, a clinical study showed that okra did not have any interaction with common oral hypoglycemic agents, such as metformin, pioglitazone sulfonylurea, and sitagliptin [111]. Understanding these interactions could facilitate the development of functional foods or health supplements that utilize okra as a key ingredient, ultimately aiding in the prevention of chronic diseases and improving overall health outcomes.

In summary, both preclinical and clinical studies support the notion that daily consumption of okra possesses beneficial biological activities for human health. Further studies are encouraged to study active components from different parts of okra, unveil new pharmacological effects (e.g., IBD and mastitis), and evaluate its efficacy in different diseases in clinical settings for the development of functional foods or health supplements aimed at promoting public health and preventing chronic diseases.

## Figures and Tables

**Table 1:** Summary of nutrients in okra.

Constituents	Reference
Carbohydrates	[22]
Protein	[22]
Dietary fiber	[22]
Starch	[22]
Sugar	[22]
Fat	[22]
Total omega-3 fatty acids	[22]
Total omega-6 fatty acids	[22]
Calcium	[22]
Phosphorus	[22]
Magnesium	[22]
Copper	[22]
Selenium	[22]
Manganese	[22]
Zinc	[22]
Sodium	[22]
Iron	[22]
β-carotene	[23]
Nicotinic Acid	[23]
Riboflavin	[23]
Thiamine	[23]
Vitamin A	[23]
Vitamin C	[23]
Vitamin K	[23]
Vitamin B complex	[23]

**Table 2:** Summary of active components in okra.

Compound Name	Class	Biological Activity	Isolated from Part of the Plant	References
Quercetin 3-O-glucosyl (1→6) glucoside (QDG)	Flavonoids	Antioxidant, hepatoprotective	Seed	[26]
Quercetin-3-O-gentiobiose	Flavonoids	Antioxidant and antifatigue Antidiabetic Vasoprotective	Pod	[31,32,33]
Isoquercitrin = quercetin 3-O-glucoside (QG).	Flavonoids	Antioxidant Antifatigue Anticancer Antidiabetic Antihyperlipidemic Hepatoprotective	Pod and seed	[25,26,31,34]
Rutin	Flavonoids	Antioxidant Antidiabetic Neuroprotective	Pod	[30,32]
Quercetin	Flavonoids	Neuroprotective	Pod	[30]
Quercetin-3-gentiobioside	Flavonoids	Antitumor	Pod	[35,36]
Quercetin-3-sambubioside	Flavonoids	Antitumor	Pod	[36]
Quercetin-3-malonylglucoside	Flavonoids	Antitumor	Pod	[36]
Catechin	Flavonoids	Antioxidant	Pod	[37]
Epicatechin	Flavonoids	Antioxidant	Pod	[37]
Proanthocyanidins: oligomeric (epi)gallocatechin	Flavonoids	Antidiabetic	Seed	[38]
Procyanidin B1	Flavonoids	Antioxidant	Seed	[37]
Procyanidin B2	Flavonoids	Antioxidant	Seed	[37]
5,7,3′,4′-tetrahydroxy flavonol-3-O-[β-D-glucopyranosyl-(1→6)]-β-D-glucopyranoside	Flavonoids	Antioxidant	Pod	[27]
5,7,3′,4′-tetrahydroxy-4″-O-methyl flavonol -3-O-β-D-glucopyranoside	Flavonoids	Antioxidant	Pod	[27]
Pectic polysaccharide AeP-P-2	Polysaccharide	Antioxidant Neuroprotective	Pod	[39]
Pectic polysaccharide WOP-2	Polysaccharide	Antidiabetic	Pod	[40]
Pectic rhamnogalacturonan	Polysaccharide	Antitumor	Pod	[41]
Water soluble pectin	Polysaccharide	Antifatigue	Stem	[42]
Pectin OP-1	Polysaccharide	Antihyperlipidemic Hepatoprotective	Pod	[43]
Water-soluble polysaccharide	Polysaccharide	Antioxidant	Pod	[44]
Acid-soluble pectin	Polysaccharide	Antiinflammatory Antioxidant	Pod	[45]
Polysaccharide OFPS11	Polysaccharide	Antiinflammatory	Flower	[46]
Polysaccharide AP1-b	Polysaccharide	Antiinflammatory	Pod	[47]
Acidic soluble polysaccharide	Polysaccharide	Antimicrobial	Pod	[48]
Polysaccharide	Polysaccharide	Antihyperlipidemic Antidiabetic	Pod	[49]
Rhamnogalacturonan	Polysaccharide	Antidiabetic Antimicrobial	Pod	[28,50]
Protein hydrolysate	Protein	Antioxidant Antidiabetic Antihyperlipidemic	Seed	[51]
Lectin	Protein	Antitumor Anti-inflammatory Antinociceptive	Seed Pod	[29,52,53]
Soluble dietary fiber	Dietary fiber	Antidiabetic	Pod	[54]
Abscisic acid	Plant hormones	Antidiabetic	Pod	[55]
Linoleic acid	Fatty acids	Antioxidant	Seed	[56]
Oleic acid	Fatty acids	Antioxidant	Seed	[56]
Palmitic acid	Fatty acids	Antimicrobial	Pod	[24]
Stearic acid	Fatty acids	Antimicrobial	Pod	[24]

**Table 3:** Summary of therapeutic effects of okra in in vivo experiments.

Type of Therapeutic Effects	Type of Experiments	Testing Subjects	Description of the Effects	References
Antidiabetic effect				
➢ Restoration of β-cell function	In vivo	SD rats	↓ Exacerbation of β islets → ↓ HbA1, HOMA-IR, and serum glucose levels.	[60]
	In vivo	Female Wistar rats	↓ PPAR-α and –γ mRNA in pancreas → ↑ β-cell in large and small islet in pancreas and ↑ reduced islet’s size, pancreatic disruption, and vacuolization.	[61]
	In vivo	Male Wistar rats	↓ Pancreatic beta cell damage, also contain oxidative factors → repair beta cell and ↑ insulin levels.	[62]
➢ Improving insulin resistance/sensitivity/glucose tolerance	In vivo	Female Wistar rats	↓ PPAR-α and –γ mRNA in pancreas → ↓ HOMA-IR, fasting blood glucose, and ↑ serum insulin.	[61]
	In vivo	Female C57BL/6 mice	↓ PPAR-α and –γ mRNA expression in liver, → ↓ HOMA-IR, blood glucose, fasting blood glucose, and serum insulin.	[25]
	In vivo	C57BL/6 mice	↓ PPAR-α, -γ and –β/δ mRNA expression in adipose tissue → ↓ blood glucose and ↑ insulin sensitivity and glucose tolerance.	[49]
	In vivo	Male Wistar rats	↓ PTP1B and PPAR-α expressions in liver tissues →↓ HOMA-IR, blood glucose, and fasting blood glucose.	[62]
	In vivo	Male Wistar rats	↑ AMPK-α activation, ↓ PEPCK ex-pression → ↑ insulin level → ↑ insulin sensitivity.	[63]
➢ Antioxidant activity	In vivo	Male Wistar albino rats	↑ SOD, CAT, GPx, and GSH levels and ↓ lipid peroxidation (TBARS) in liver, kidney, and pancreases. ↓ Blood glucose.	[58]
	In vivo	Male Wistar rats	↑ Erythrocyte GSH level and FRAP content. ↓ Erythrocyte PMRS activity. ↓ Erythrocyte MDA and plasma AOPP.	[64]
	In vivo	Male ICR mice	↓ Fasting blood glucose and serum MDA. ↑ SOD activity and serum insulin levels.	[40]
➢ Gestational diabetes	In vivo	Female and male SD rats	↑ SOD, GPx, GSH, and CAT content in liver and pancreas → ↓ fasting blood glucose, HbA1c, fasting insulin, and ↑ hepatic glycogen.	[65]
➢ Inhibition of rate of carbohydrate digestion and glucose absorption	In vivo	Long Evans rats	↓ Glucose absorption → ↓ blood glucose level.	[54]
➢ Hypoglycemia	In vivo	Male Wistar albino rats	↓ Blood glucose level.	[66]
	In vivo	Male Wistar albino rats	↓ Blood glucose level and HbA1c.	[67]
	In vivo	Male C57BL/6 mice	↓ Blood glucose level and glucose tolerance.	[28]
	In vivo	Male SPF grade C57BL/6 mice	↓ Fasting blood glucose level.	[68]
➢ Diabetic nephropathy	In vivo	Male SD rats	↓ Urine albumin excretion → improve renal function. ↓ Creatinine clearance rate → ↓ hyperfiltration → improve renal function. ↓ Matrix deposition → ↓ renal fibrosis. ↓ Kidney DPP-4 and ↑ GLP-1R expression. ↓ Serum and kidney TBARS.	[69]
➢ Restoration of diabetic-induced splenic damage	In vivo	Male Wistar rats	↓ Reduction of white pulp, ↑ active red pulp, and ↑ hemosiderin deposition → ↑ effect on restoring the normal immunological function of the spleen.	[70]
Antifatigue effect	In vivo	Male Kunming mice	↑ Weight-loaded swimming endurance time. ↑ HG content. ↓ SUN and BLA content.	[57]
	In vivo	Male Kunming mice	↑ SDH, ATP, and ATPase levels and ↓ LDH and CK levels → ↑ swimming time, ↓ SUN and BLA content, and ↑ HG and MG content.	[71]
	In vivo	Male ICR mice	FRAP and reducing power as well as ↓ hepatic MDA and ↑ SOD and GSH-Px → ↑ swimming time, ↓ BLA and SUN content, and ↑ HG content.	[31]
	In vivo	Male SD rat	↑ Swimming endurance time. ↓ BLA, SUN, and MDA levels. ↑ HG, MG, SOD, and GSH-Px levels.	[33]
Vasoprotective effect	In vivo	Male SD rat	↓ Serum MDA level. ↑ SOD and GSH-Px levels → ↓ serum MCP-1, IL-6, and TNF-α levels. ↓ Ox-LDL, LOX-1, and NF-κB p65 expression in aortic tissues. ↓ Ox-LDL, LOX-1, and mRNA expression in aortic tissues → endothelial dysfunction ↓ foam cell in aorta, aorta thickness, and intima–medial thickness.	[33]
Hepatoprotective effect				
➢ Antioxidant activity	In vivo	Male Wistar rats	↑ Hepatic CAT, SOD, and GSH in rats → ↓ hepatic TG, MDA, and TNF-α, serum AST, ALT, ALP, and total bilirubin content in rats, ↑ serum Albumin in rats, as well as ↓ steatosis, inflammation, and necrosis in rat liver.	[72]
	In vivo	Wistar albino rats	↓ Serum GOT, GPT, ALP, and GGT levels. ↓ Serum TC and TG levels. ↓ Hepatic MDA and non-protein sulfhydryls (NP-SH) and total protein (TP). ↓ Liver inflammation.	[73]
Antihyperlipidemia effect	In vivo	Female Wistar rats	↓ PPAR-α and –γ mRNA in pancreas → ↓ serum TG and TC.	[61]
	In vivo	Female C57BL/6 mice	↓ PPAR-α and -γ and aP2 mRNA expression in liver → ↓ TG → ↓ hepatic steatosis.	[25]
	In vivo	C57BL/6 mice	↓ PPAR-α, -γ, -β/δ, and UCP2. mRNA expression in adipose tissue and LXR and its target ABCG1, ApoE, CYP7A1, and LPL mRNA expression in liver → ↓ serum TC, LDL-c, and ↑ HDL-C. ↓ Size of white adipocytes.	[49]
		Mice white adipocytes tissue		
	In vivo	SD rats	↓ TG and FFA. ↑ HDL/LDL ratio and HDL.	[60]
	In vivo	Male Wistar albino rats	↓ TC, TG, LDL, and VLDL. ↑ HDL.	[67]
	In vivo	ddY mice	↓ Serum TC and TG.	[74]
	In vivo	Male C57BL/6J mice	↑ CYP7A1 mRNA expression and ↓ SREBP1c and FAS mRNA expression → ↓ serum TG, TC non-HDL-C, non-HDL-C/HDL-C, and hepatic TG, TC, and ↑ fecal bile acid (bile acid excretion).	[75]
Antitumor activity				
➢ Immunomodulatory activity	In vivo	BALB/c inbred mice	↑ Serum TNF-α, IFN-γ, and ↓ IL-10 levels in mice. ↑ Thymus and spleen index and ↑ splenocyte proliferation in mice.	[76]
Neuroprotective effect	In vivo	Adult male Swiss albino mice	↓ Step-down latency → memory impairment. ↓ Acute restraint stress-induced change in biochemical parameters, e.g., plasma corticosterone, TC, TG, and glucose. ↓ Immobility time. ↑ Time spent and number of entries in open arms of elevated plus arms.	[77]
	In vivo	Male Swiss albino mice	↓ Duration of immobility in forced swimming test and tail suspension tests → antidepressant activity.	[78]
	In vivo	Male ICR mice	↓ Escape latency time and ↑ time spent om target quadrant → ↑ learning and ↓ memory impairment. ↑ NR2A/B protein expression. ↑ Average number of BrdU-positive cell per section → ↑ dentate gyrus cell proliferation. ↑ Number of CA3 hippocampal neurons and ↓ morphological damage in the CA3 region.	[30]
	In vivo	Male Wistar rat	↓ Malondialdehyde level and ↓ matrix membrane metalloproteinase-9 level.	[79]
Skin protective effect	In vivo	Normal women	↑ Skin elasticity, firmness, texture, density and ↓ wrinkle in vivo.	[80]
Anti-temporomandibular joint (TMJ) inflammatory hypernociception				
➢ Anti-inflammation	In vivo	Swiss albino mice	↓ Carrageenan induced paw edema.	[81]
	In vivo	Wistar rats		[52]
	In vivo	Male Wistar rats	↓ TNF-αand IL-1βand ↑ HO-1 expression in TMJ tissue → ↓ TNF-α and IL-1β in TMJ tissue and trigeminal ganglion. ↓ Leukocyte cells, MPO activity, and evans blue extravasation in TMJ synovial lavage. ↓ Inflammatory cell influx (↓ inflammatory cell and edema in synovial membrane.	[82]
	In vivo	Male Wistar rats	↓ Evans blue extravasation. ↓ TNF-α in TMJ tissue, trigeminal ganglion, and subnucleus caudalis.	[83]
➢ Analgesic activity	In vivo	Swiss albino mice	↓ Acetic acid induced writhing.	[81]
	In vivo	Male Swiss albino mice	↓ Acetic acid induced abdominal writhing.	[52]
➢ Antinociceptive activity	In vivo	Swiss albino mice	↓ Licking activity.	[81]
	In vivo	Male Wistar rats	↑ Head withdrawal threshold → ↓ mechanical hypernociception.	[82]
	In vivo	Male Wistar rats	Activation of central opioid receptors (δ and κ but not µ) → ↓ nociceptive behavior.	[83]
Anti-gastric ulcer effect				
➢ Gastroprotective effect	In vivo	Male Wistar rats	↓ Ulcer formation. ↓ Blood MDA and GSH levels. ↑ Serum β—carotene and retinol levels. ↑ PCNA-positive nuclei marker → ↑ cell proliferation in gastric mucosal healing area. ↓ TUNEL positive apoptotic cell. ↓ Gastric damage (↓ edema, hemorrhage, and inflammation scores).	[84]
Antidepressive effect				
➢ Anti-inflammatory effect	In vivo	Male C57BL/6 mice	↓ Toll-like receptor 4 (TLR4)/NF-κB, ↓ NLRP3 inflammasome, and Akt/PI3K pathways, →↓ inflammation. ↑ Activation of MAPK pathways →↑ anti-inflammatory effect → the bidirectional communication of microbiota-gut-brain axis via regulation of inflammation response.	[85]

Key: ↑ = activate/enhance/increase; ↓ = decrease/inhibit/reduce; → = lead to.

**Table 4:** Summary of therapeutic effects of okra in in vitro experiments.

Type of Therapeutic Effects	Type of Experiments	Testing Subjects	Description of the Effects	References
Antidiabetic effect				
➢ Restoration of β-cell function	In vitro	RINm5F cell	↓ % subG1. ↓ Procaspase and caspase 3, DPP-4, AMPK, and Bax expression. ↑ GLP-1R, mTOR, and PI3K expression. ↓ apoptosis.	[86]
➢ Antioxidant activity	In vitro	N.A.	Good antioxidant activity in DPPH, ABTS, and FRAP.	[25]
	In vitro	N.A.	Good antioxidant activity in DPPH and FRAP.	[87]
	In vitro	N.A.	High antioxidant activity in DPPH and ABTS.	[37]
	In vitro	N.A.	Strong antioxidant activity in DPPH and FRAP.	[27]
	In vitro	N.A.	High scavenging activity on superoxide and hydroxyl radical.	[40]
	In vitro	N.A.	Good antioxidant activity in DPPH.	[62]
➢ Inhibition of rate of carbohydrate digestion and glucose absorption	In vitro	α-glucosidase and α-amylase	↓ Activity of α-glucosidase and α-amylase.	[38,88]
	In vitro	Diffusion system	↓ Glucose diffusion.	[89]
➢ Diabetic nephropathy	In vitro	HK-2	↓ Vimentin, AT-1, TGF-β1, and DPP-4 expression. ↑ cadherin expression.	[90]
Antifatigue effect	In vitro	N.A.	Good antioxidant activity in DPPH, FRAP, and reducing power.	[31]
Hepatoprotective effect				
➢ Antioxidant activity	In vitro	N.A. HepG2	High in DPPH, hydroxy radical scavenging activity, and total antioxidant capacity. ↑ GSH in HePG2 and → ↓ ALT, AST, and MDA in HepG2.	[72]
	In vitro	N.A.	Strong reducing power and DPPH, superoxide, and hydroxyl radical scavenging activity ↓ MDA content. ↓ GPT and GOT activity. ↑ SOD and CAT activity.	[26]
	In vitro	BRL-3A		
➢ Antilipotoxicity activity	In vitro	HepG2 cells	↓ OA-induced lipid accumulation, ROS formation, apoptosis, leakage of transaminases, and inflammatory cytokine secretion →↓ lipotoxicity. ↑ Activation of Adenosine 5′-monophosphate (AMP)-activated protein kinase pathway → ↓ lipotoxicity.	[43]
Antihyperlipidemia effect	In vitro	N.A.	High bile acid binding capacity.	[75]
➢ Antilipotoxicity activity	In vitro	HepG2 cells	↓ OA-induced lipid accumulation, ROS formation, apoptosis, leakage of transaminases, and inflammatory cytokine secretion →↓ lipotoxicity. ↑ Activation of Adenosine 5′-monophosphate (AMP)-activated protein kinase pathway → ↓ lipotoxicity.	[43]
Antitumor activity				
➢ Antiproliferation and apoptosis	In vitro	MCF7 and CCD-1059 sk	↓ Cell growth % in MCF7 but not CCD-1059 sk. ↑ Caspase-3 and -9 mRNA expression. ↑ p21 mRNA expression and BAX/Bcl-2 expression. ↓ Bcl-2 mRNA expression → ↑ apoptosis in MCF7. ↑ Necrosis in MCF7 depend on interaction with cell surface-expressed carbohydrates.	[29]
	In vitro	Highly metastatic B16F10	↓ Proliferation indices and ↑ % apoptosis cells. ↑ % of cells in G2/M and ↓ % of cells in G1. ↓ Cadherins and α5 integrin expression. ↑ Gal-3 expression.	[41]
➢ Immunomodulatory activity	In vitro	BMHC-imDCs	↑ Cell size, polymorphic nuclei, dendritic protrusions → ↑ dendritic cell maturation. ↑ MHC class II and CD80/86 expression on the cell surface. ↓ endocytosis activity. ↑ IL-12, IFN-γ, and ↓ IL-10 level → ↑ TH1 response.	[91]
	In vitro	HepG2 and RAW 264.7	↑ NF-κB p65 expression → ↑ iNOS expression and iNOS and TNF-α mRNA expression. ↑ NO, TNF-α, and IL-1β levels. ↑ Phagocytic activity of macrophage. ↑ Macrophage response → ↓ proliferation of HepG2.	[46]
	In vitro	RAW 264.7	↑ RAW 264.7 proliferation. ↑ iNOS expression in RAW 264.7 → ↑ NO level. ↑ TNF-α, IFN-γ, and IL-10 levels in RAW 264.7.	[76]
Neuroprotective effect	In vitro	N.A.	Good antioxidant activity in FRAP, DPPH, β-Carotene-Linoleic acid, and good chelating effect on ferrous ions.	[77]
	In vitro	SH-SY5Y (wild type and H63D HFE forms)	↓ Protein carbonyl l, H_2_O_2_, and intracellular ROS levels in cells. ↓ Tau ps199, 202, and 396, and GSK-3β expression. ↓ Intracellular iron in cells.	[92]
Skin protective effect	In vitro	Fibroblast	↑ Protection % of FGF-2 placed in physiological conditions and concentration of FGF-2 in cells. ↑ Sulphated GAG synthesis in fibroblast. ↑ Fibroblast cell proliferation.	[80]
		N.A.	Good antioxidant capacity in DPPH, ABTS, and FRAP. ↓ UV-B radiation induced cytotoxicity, DNA damage (nongenotoxic), as well as loss of cell membrane integrity and apoptosis. ↓ Nrf2 and HO-1 protein and mRNA expression → ↓ intracellular ROS and depletion of SOD, CAT, GPx, and GR.	[93]
	In vitro	HDF		
Anti-gastric ulcer effect				
➢ Anti-adhesive effect of *H. pylori* to gastric mucosa	In vitro	*H. pylori* and human gastric mucosa	Interactions of compounds from okra with bacterial surface structure → ↓ adhesion of *H. pylori* in human gastric mucosa.	[48]
	In vitro	*H. pylori* and human gastric epithelia AGS cell	↓ Bacteria binding to SabA, laminin, lactoferrin, BabA, and HpA binding site → ↓ Adhesion of *H. pylori* in human gastric epithelia AGS cells. Esterification → ↑ anti-adhesive activity.	[50]
	In vitro	*H. pylori* and human adherent gastric adenocarcinoma epithelia cells	↓ binding to BabA, SabA, and fibronectin binding adhesin → ↓ adhesion of *H. pylori* in AGS.	[94]
	In vitro	*H. pylori*	*H. pylori* strains with HopQ genotype or CagA → ↓ adhesion activities.	[95]
Antimicrobial activity				
➢ Antibacterial activity	In vitro	*Bacillus cereus* and *Micrococcus flavus* *Staphylococcus aureus*, *Listeria monocytogenes*, *Escherichia coli*, *Enterobacter cloacaea*, *Salmonella enteritidis*, and *S. typhimurium*	Bacteriostatic activity of different genotypes of okra were lower than streptomycin but comparable to ampicillin especially *Listeria monocytogenes*, *Salmonella typhimurium*, and *Salmonella enteritidis.*	[96]
	In vitro	*Rhodococcus erythrolis R. opacus*, *Mycobacterium* sp., *M. aurum*, *Staphylococcus aureus*, *Escherichia coli*, *Xanthobacter Py2*, and *Pseudomonas aeruginosa*	Low minimum inhibitory concentration against *S.aureus*, *Mycobacterium* sp., *Mycobacterium aurum*, and *X*. Py2. Large inhibition area on the above-mentioned bacteria strains. ↓ Cell viability of bacterial strains.	[24]
	In vitro	*H. pylori* strains	Had zone of inhibition → susceptible to okra. Moderately high MIC. Showed time dose-dependent bactericidal effect.	[97]
➢ Antifungal activity	In vitro	*Aspergillus fumigatus*, *A.versicolor, A. ochraceus*, *A. niger*, *Cladosporium cladosporioides*, *Penicillium funiculosum*, and *P. verrucosum*	Different genotypes of okra showed better or comparable fungistatic and fungicidal activity than ketoconazole, while bifonazole was much more effective than them.	[96]

Key: ↑ = activate/enhance/increase; ↓ = decrease/inhibit/reduce; → = lead to.

**Table 5:** Summary of clinical studies on okra.

Study Design	Subjects	Intervention	Description of the Findings	References
Randomized, double-blind, placebo-controlled clinical trial	94 patients with type II diabetes (aged 40–60) in Iran	Treatment: 1000 mg powdered okra thrice per day for 3 months Placebo: with the same dosage	Improved glycemic control: ↓ hba1c, fasting blood glucose (FBG), HOMA-IR, and insulin levels Improved hyperlipidemia: ↓ TG and TC Alleviated inflammation: ↓ high-sensitivity C-reactive protein (hs-CRP) No reported adverse effects	[113]
Randomized double-blinded, single-center, plcebo-controlled clinical trial	48 patients with type II diabetes (aged 30–75) in Iran	Treatment: 10 g okra powder (equivalent to 100 g fresh okra) blended in 150 g yogurt (twice per day lunch and dinner) for 8 weeks Placebo: yogurt with consumable color	Improved glycemic control: ↓ Fasting plasma glucose (FPG), HOMA-IR, and ↑ Quantitative insulin sensitivity checkindex (QUICKI TC, TG LDL-C, LDL-C/ HDL-C ratio No reported adverse effects	[108]
Randomized, non-blinded controlled trial	60 women with gestational diabetes mellitus (aged 18–35) in Iran	Treatment: 3 g of okra skin and seed powder twice per day for 4 weeks. Control: intervention	Improved glycemic control after 2- and 4-week consumption: ↓ fbg and postprandial blood glucose (ppg)	[112]
Clinical trial	40 patients with type II diabetes and hypercholesterolemia (aged 45–65) in Indonesia	Treatment 1: 40 g boiled okra per day for 2 weeks Treatment 2: 40 g stream okra per day for 2 weeks Control: no intervention	Improved glycemic control (both treatments): ↓ fbg	[107]
Randomized, double-blinded, placebo-controlled clinical trial	70 patients with pre-diabetes (aged 30–55) in Iran	Treatment: 2 capsules of 500 mg okra (composed with okra powder + magnesium stearate in 10 to 1 ratio) thrice per day for 8 weeks Placebo: 2 capsules of 500 mg placebo capsules (composed of carboxymethyl cellulose + magnesium stearate in 10 to 1 ratio) thrice per day for 8 weeks	Improved hyperlipidemia: ↓ TC, LDL-C, and ↑ HDL-C Reduced liver and kidney damage: ↓ ALT, AST, and uric acid No side effect	[105]
Randomized, double-blind, placebo-controlled clinical trial	99 patients with diabetes (aged above 18) receiving oral hypoglycemic medications in Iran	Treatment: 1000 mg powdered okra capsule every 6 h for 8 weeks Placebo: microcrystalline cellulose capsule every 6 h for 8 weeks	Improved glycemic control: ↓ FBG, blood sugar, and hba1c No side effect No significant effect on lipid profile	[111]
Randomized, triple-blind, placebo-controlled clinical trial	55 patients with diabetic nephropathy (aged 40–70) in Iran	Treatment: capsule containing 80 mg dried okra extract per day for 10 weeks Placebo: capsule of carboxymethylcellulose per day for 10 weeks	No significant effect on renal function indices, lipid profile, and inflammation	[106]
Randomized, triple-blind, placebo-controlled clinical trial	55 patients with diabetic nephropathy (aged 40–70) in Iran	Treatment: capsule containing 80 mg dried okra extract per day for 10 weeks Placebo: capsule of carbox-ymethylcellulose per day for 10 weeks	↓ Energy and carbohydrate intake	[109]
Randomized, triple-blind, placebo-controlled clinical trial	55 patients with diabetic nephropathy (aged 40–70) in Iran	Treatment: capsule containing 80 mg dried okra extract per day for 10 weeks Placebo: capsule of carbox-ymethylcellulose per day for 10 weeks	Improved glycemic control: ↓FBG, HOMA-IR, and hba1c (in treatment group but not significant between group) No significant effect on renal function, inflammation	[110]
Randomized, double-blind, three-armed, placebo-controlled clinical trial	101 overweight to moderately obese adults (aged 18–65) in Germany	Treatment 1: high dose IQP-AE-103 (330 mg dehydrated okra powder and 85 mg inulin) thrice per day after meal for 12 weeks Treatment 2: low dose IQP-AE-103 (165 mg dehydrated okra powder and 42.5 mg inulin) for 12 weeks Placebo: capsules containing standard excipients for 12 weeks	Improved anthropometric measures ↓ weight loss, BMI, waist circumference, and hip circumference (both dosage of IQP-AE-103) ↓ Body Fat ↓ Feeling of hunger in 66% subjects (high dosage) No side effects reported	[114]

Key: ↑ = activate/enhance/increase; ↓ = decrease/inhibit/reduce.

## Data Availability

No new data were created or analyzed in this review. Data sharing is not applicable to this article.

## List of Abbreviations

Abbreviations	Definitions
ABCG1	ATP-binding cassette transporter G1
ABTS	2,2′-azino-bis(3-ethylbenzothiazoline-6-sulfonic acid
AE	*Abelmoschus esculentus*
AIF	Apoptosis-inducing factor
ALP	Alkaline phosphatase
ALT	Alanine transaminase
Akt	Protein kinase B
AMP	Adenosine 5′-monophosphate
AMPK	Adenosine monophosphate-activated protein kinase
AOPP	Advanced oxidation protein products
aP2	Adipocyte protein 2
ApoE	Apolipoprotein E
AST	Aspartate transaminase
AT-1	Angiotensin II receptor-1
ATPase	Adenosine 5′-TriPhosphatase
Bax	B-cell lymphoma protein 2 associated X
Bcl-2	B-cell lymphoma 2
BLA	Blood lactic acid
BMHC-imDCs	Rat bone marrow hematopoietic cells derived immature dendritic cells
BrdU	Bromodeoxyuridine
CA3	Cornu Ammonis 3
CAT	Catalase
CCl_4_	Carbon tetrachloride
CD	Cluster of differentiation
CK	Creatine kinase
CYP7A1	Cytochrome P450 7A1
DCs cell	Dendritic cells
DPP-4	Dipeptidyl peptidase-4
DPPH	2,2-Diphenyl-1-picrylhydrazyl
EMT	Epithelial-mesenchymal transition
FAS	Fatty acid synthase
FGF-2	Fibroblast growth factor-2
FRAP	Ferric reducing ability of plasma
FST	Forced swimming test
GAG	Glycosaminoglycans
Gal-3	Galectin-3
GGT	Gamma glutamyltransferase
GLP-1R	Glucagon like peptide-1 receptor
GOT	Glutamate oxaloacetate transaminase
GPT	Glutamate pyruvate transaminase
GPx	Glutathione peroxidase
GR	Glutathione reductase
GSH	Glutathione
GSH-Px	Glutathione peroxidase
GSK-3β	Glycogen synthase kinase-3 beta
HbA1c	Glycated hemoglobin
HDF	Human dermal fibroblast adult cell
HDL	High-density lipoprotein
HDLC	High-density lipoprotein-cholesterol
HFE	Hemochromatosis protein
HG	Hepatic glycogen
HO-1	hemeoxygenase-1
HOMA-IR	Homeostasis model assessment of insulin resistance
ICAM-1	Intercellular adhesion molecule-1
IFN-γ	Interferon gamma
IL-6	Interleukin-6
IBD	Inflammatory bowel disease
iNOS	Inducible nitric oxide synthase
LDH	Lactate dehydrogenase
LDL	Low-density lipoprotein
LDL-c	Low-density lipoprotein-cholesterol
LOX-1	Lectin-like oxidized low-density lipoprotein receptor 1
LPL	Lipoprotein lipase
LXR	Liver X receptors
MAPK	Mitogen-activated protein kinase
MCP-1	Monocyte chemoattractant protein-1
MDA	Malondialdehyde
MG	Muscle glycogen
MHC	Major histocompatibility complex
MIC	Minimum inhibitory concentration
MPO	Myeloperoxidase
mRNA	Messenger ribonucleic acid
mTOR	Mammalian target of rapamycin
NAFLD	Non-alcoholic fatty liver disease
NF-κB	Nuclear transcription factor-κB
NLRP3	Nucleotide-binding domain and leucine-rich repeat containing family Pyrin domain containing 3
NMDA	N-methyl-D-aspartate
NO	Nitric oxide
Non-HDLC	Non-high-density lipoprotein-cholesterol
NR	NMDA-receptor
Nrf2	Nuclear factor E2-related factor-2
OA	Oleic acid
Ox-LDL	Oxidized low-density lipoprotein
PCNA	Proliferating cell nuclear antigen
PI3K	Phosphoinositide 3-kinase
PMRS	Plasma membrane redox system
PPAR	Peroxisome proliferator-activated receptor
PTP1B	Protein tyrosine phosphatase 1B
RG-I	Rhamnogalacturonan-I
SDH	Succinate dehydrogenase
SOD	Superoxide dismutase
SREBP1c	Sterol regulatory element-binding protein 1c
SUN	Serum urea nitrogen
TBARS	Thiobarbituric acid reactive substances
TC	Total cholesterol
TG	Triglyceride
TGF-β1	Transforming growth factor β1
TH1	Type 1 T helper
TMJ	Temporomandibular joint
TNF-α	Tumor necrosis factor alpha
TLR4	Toll-like receptor 4
TUNEL	Terminal deoxynucleotidyl transferase dUTP nick end labeling
UCP2	Uncoupling protein 2
UV-B	Ultraviolet B radiation
VLDL	Very-low-density lipoprotein
